# Melatonin Activation by Human Cytochrome P450 Enzymes: A Comparison between Different Isozymes

**DOI:** 10.3390/molecules28196961

**Published:** 2023-10-06

**Authors:** Thirakorn Mokkawes, Tamar De Visser, Yuanxin Cao, Sam P. De Visser

**Affiliations:** Department of Chemical Engineering, Manchester Institute of Biotechnology, The University of Manchester, 131 Princess Street, Manchester M1 7DN, UK

**Keywords:** cytochrome P450, enzyme catalysis, enzyme mechanism, computational modelling, density functional theory, molecular dynamics

## Abstract

Cytochrome P450 enzymes in the human body play a pivotal role in both the biosynthesis and the degradation of the hormone melatonin. Melatonin plays a key role in circadian rhythms in the body, but its concentration is also linked to mood fluctuations as well as emotional well-being. In the present study, we present a computational analysis of the binding and activation of melatonin by various P450 isozymes that are known to yield different products and product distributions. In particular, the P450 isozymes 1A1, 1A2, and 1B1 generally react with melatonin to provide dominant aromatic hydroxylation at the C_6_-position, whereas the P450 2C19 isozyme mostly provides *O*-demethylation products. To gain insight into the origin of these product distributions of the P450 isozymes, we performed a comprehensive computational study of P450 2C19 isozymes and compared our work with previous studies on alternative isozymes. The work covers molecular mechanics, molecular dynamics and quantum mechanics approaches. Our work highlights major differences in the size and shape of the substrate binding pocket amongst the different P450 isozymes. Consequently, substrate binding and positioning in the active site varies substantially within the P450 isozymes. Thus, in P450 2C19, the substrate is oriented with its methoxy group pointing towards the heme, and therefore reacts favorably through hydrogen atom abstraction, leading to the production of *O*-demethylation products. On the other hand, the substrate-binding pockets in P450 1A1, 1A2, and 1B1 are tighter, direct the methoxy group away from the heme, and consequently activate an alternative site and lead to aromatic hydroxylation instead.

## 1. Introduction

Due to its substantial natural abundance on Earth, iron is a common element in metalloenzymes. The mononuclear iron-containing enzymes are categorized into two primary classes based on co-factor, namely, the non-heme iron [1,2,3,4,5,6,7] and heme-iron enzymes [8,9,10,11,12,13,14,15,16,17,18]. The heme-iron enzymes are further subdivided into heme monooxygenases, heme-catalases, heme-peroxidases and heme-dioxygenases based on their use of either O_2_ or H_2_O_2_ and the number of oxygen atoms that are transferred to the substrate. In this work, we focus on a special class of heme-monooxygenases, namely the cytochromes P450. These are important enzymes for human health, catalyze the majority of xenobiotic metabolism processes in the liver [19,20,21] and, in addition, are involved in hormone biosynthesis reactions including estrogen [22]. Despite years of scientific studies on the structure and catalytic mechanism of P450 enzymes, as well as their characterization, several key questions regarding P450 catalysis remain unanswered.

**Figure 1:**
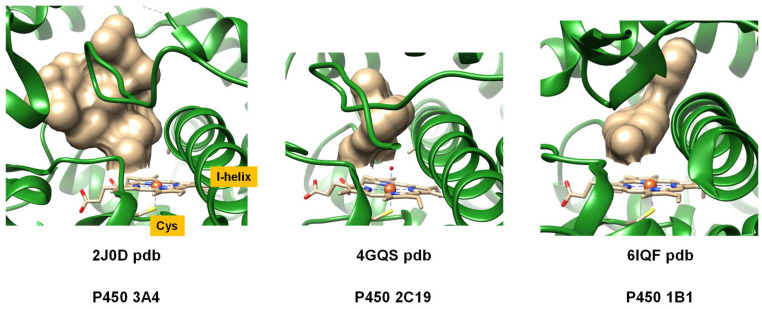
Extracts of the active site of three P450 isozymes, namely P450 3A4, P450 2C19 and P450 1B1.

P450 isozymes are ubiquitous, and are found in nearly every form of life. To date, over 21,000 structures have been determined [23,24] and subsequently categorized into families and subfamilies based on structural homology and chemical functionality. While all P450 isozymes centrally incorporate an iron atom within a protoporphyrin IX group of a heme scaffold, they exhibit variations in overall protein architecture and folding. Diverse functional differences are evident among P450 isozymes, especially in the areas of substrate-binding, reactivity, selectivity, and catalytic turnover. To illustrate these structural variations, Figure 1 highlights extracts of P450 isozymes with a focus on the active site as obtained from the protein databank (pdb) [24] for three representative human P450 isozymes, namely, the P450 isozymes 3A4 (2J0D pdb [25]), P450 1B1 (6IQ5 pdb [26]), and P450 2C19 (4GQS pdb [27]). In each structure, the bound substrate (highlighted in grey spheres) is located on the distal site of the heme, adjacent to the I-helix of the protein. Upon close inspection of these structures, it is evident that all P450 isozymes possess analogous structural traits, including comparable helices constituting their secondary structure. However, the constituent amino acids in these chains exhibit considerable variations, which, in turn, affect the dimension, shape and form of the substrate binding pocket as well as the hydrophobicity of the active site environment. As a consequence, different P450 isozymes activate substrates differently, thereby leading to changes in the types of products obtained and the product distributions.

**Figure 2:**
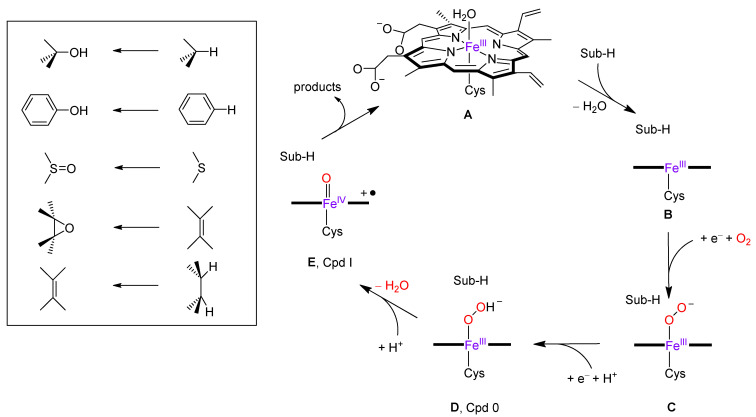
Catalytic cycle of P450 enzymes and products obtained of oxygen atom reactions.

The catalytic cycle of P450 enzymes is well-documented and several transient species in the cycle have been trapped and characterized [8,9,10,11,12,13,14,15,28,29,30,31,32,33]. In the resting state, the heme exhibits an iron(III) or ferric oxidation state with the metal bound to the four nitrogen atoms of the heme in the equatorial plane. It also forms a bond with the thiolate group of a Cys residue in the axial position. This cysteinate axial ligand is a conserved residue in the P450 structure and has been linked with the biosynthesis and activity of the reactive species in the catalytic cycle, namely the iron(IV)-oxo heme cation radical, also referred to as Compound I (Cpd I). The opposite side of the heme with respect to the cysteinate residue is the distal site of the heme, where, in the resting state, a water molecule is bound, while, in its vicinity, the substrate positions itself in the active site. The nomenclature “P450” is derived from the Soret absorption band at 450 nm, observed in the CO-bound ferric complex [34,35]. All P450s partake in a catalytic cycle that requires two electrons from a redox partner and two protons from the solvent to produce Cpd I. Figure 2 illustrates the intricacies of the P450 enzymatic catalytic cycle, which starts from the resting state (structure **A**). The cycle is triggered by substrate binding, resulting in the release of the water molecule from the heme distal site. This step converts the ferric heme from a six-coordination to a five-coordination geometry and induces a shift from a low-spin to a high-spin state, thereby yielding structure **B**. The heme is then reduced by a redox partner (typically NADPH) to provide the pentacoordinated ferrous complex [36,37,38,39], and subsequently binds molecular oxygen (depicted as structure **C** in Figure 2). Another redox step, coupled with a swift proton transfer, generates the iron(III)-hydroperoxo complex (structure **D**), alternatively termed Compound 0 (Cpd 0). A secondary proton relay process results in the formation of the Cpd I structure (structure **E**) [40,41,42], a multifaceted oxidant that undertakes the oxygen atom transfer reaction to the substrate. The insertion of the oxygen atom into the substrate typically results in aromatic or aliphatic hydroxylation, sulfoxidation, or epoxidation [43,44,45,46,47,48,49,50,51,52,53,54,55,56,57,58,59,60,61,62,63,64,65,66,67]. However, desaturation and decarboxylation reactions have also been documented [49,68,69]. Given that bifurcation pathways in distinct P450 isozymes yield varied products and unique isozyme-specific product distributions, many scholars have probed the origins of these bifurcation pathways.

**Figure 3:**
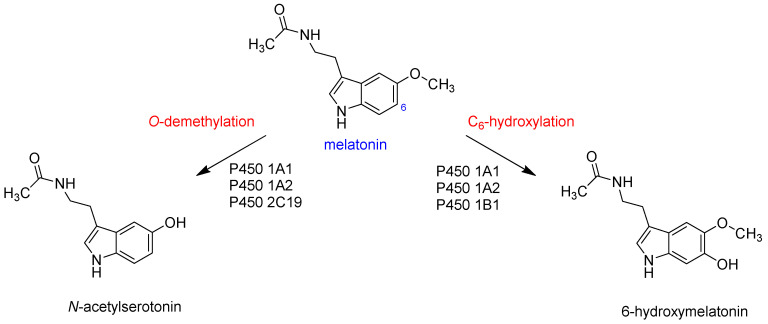
Reaction products observed for P450 activation of melatonin.

A particular biomolecule that was shown to yield varying products upon activation by human P450 isozymes is melatonin [70,71,72,73], as illustrated in Figure 3. Predominantly, two main metabolic pathways are identified in its liver metabolism, i.e., C_6_-hydroxylation and *O*-demethylation. Ma et al. [74] quantified the product distributions resulting from melatonin activation by various P450 isozymes. Their results indicated that the P450 1A1, 1A2, and 1B1 isozymes predominantly produce aromatic hydroxylation products at the C_6_ position, while the P450 2C19 isozyme specifically yields *O*-demethylation products. To understand these product distributions, various computational studies were performed that highlighted differences in substrate positioning and the size and shape of the substrate binding pocket [75,76,77]. In particular, the work focused on the comparison of melatonin activation via P450 1A1 and 1A2 isozymes. The studies highlighted differences in the substrate binding pocket and the effects of the second coordination sphere on substrate positioning, which were shown to influence the chemoselectivity for the reaction [75,76,77,78]. The researchers postulated that the C_6_-hydroxylation reaction is triggered by an electrophilic attack from Cpd I on the C_6_-position of melatonin, followed by a proton shuttle from the *ipso*-position, via one of the heme nitrogen atoms, to the oxo group, ultimately leading to phenol formation. Conversely, *O*-demethylation is expected to be initiated with hydrogen atom abstraction from the methoxy group by Cpd I, followed by rebound of the OH group to form the corresponding alcohol. The subsequent deformylation is anticipated to occur either in the solution or within the protein, and is most likely aided by a proton source, such as H_3_O^+^. These computational studies revealed that the substrate binding pocket of P450 1A2 is tighter and more compact than the one in P450 1A1, so the optimal substrate–oxidant interactions in the transition states can facilitate both reaction pathways. However, the tight substrate binding pocket in P450 1A2 prevents the ideal orientation in the transition state for attacks on the methoxy group by Cpd I and primarily results in aromatic hydroxylation products. To gain deeper insights into the binding, orientation, and subsequent activation of melatonin by P450 isozymes, we broadened our investigations on melatonin activation by human P450 isozymes, and particularly aim to understand how P450 2C19 compares to P450 1A1 and 1A2 in terms of structure and activity.

## 2. Results

### 2.1. Docking Studies

**Figure 4:**
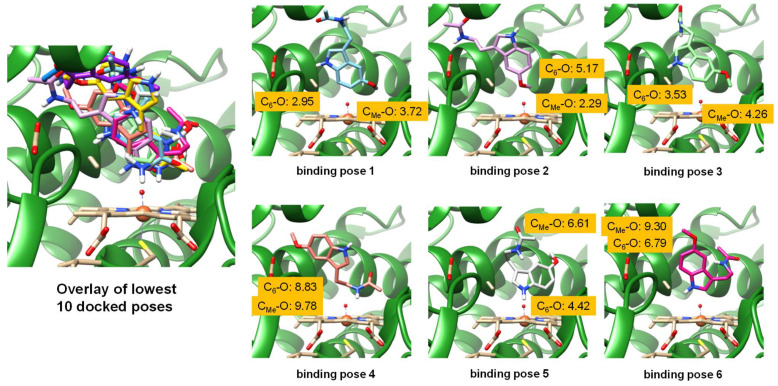
Melatonin docking into Chain A of the P450 2C19 structure of the 4GQS pdb file. Left-hand side shows the overlay of the ten lowest energy docked poses, while the individual top six poses are shown on the right, with C_6_–O and C_Me_–O distances between heme and substrate highlighted in Å.

Based on the 4GQS protein databank (pdb) file [24,27], we performed molecular docking and molecular dynamics simulations on a melatonin-bound P450 2C19 structure. We chose chain A of the pdb file and removed substrate, glycerol and water molecules from the distal bound water molecule of the heme, which was set at an initial distance of 1.63 Å and replaced by an oxo group to mimic a Cpd I-type structure. Subsequently, melatonin was docked into the structure using a box with the center of the coordinates (*x*, *y*, *z*) = (−81.52, 22.85, −45.13) and a box size of *x* = 21.57, *y* = 18.73 and *z* = 18.68 using the Autodock Vina 1.2.0. software package, as implemented in Chimera [79,80]. An overlay of the 10 lowest-energy poses is shown on the left-hand side of Figure 4. The lowest-energy poses all have the substrate bound close to the heme on the distal site, and six poses are highlighted on the right-hand side of Figure 4. As can be seen, the substrate is locked in roughly the same area of the substrate binding pocket, adjacent to the I-helix and close to the B’-helix. This area of the substrate binding pocket is mostly lined with aromatic and aliphatic amino acid residues and also includes the side chain of Asp_293_, as highlighted in Figure 4. In some binding poses, it forms a hydrogen bond with the substrate amide group.

We then measured distances from the C_6_-atom of melatonin to the oxo group of the heme (C_6_–O distance) and the distance from the carbon atom of the methoxy group to the oxo group of the heme (C_Me_–O distance) for all six binding poses highlighted in Figure 4. The lowest-energy binding pose has a C_6_–O distance of 2.95 Å and a C_Me_–O distance of 3.72 Å, and hence a shorter C_6_–O than C_Me_–O distance, which would imply preferential C_6_-hydroxylation over methoxy-hydroxylation. This disagrees with the experiment that measured preferential *O*-demethylation over C_6_-aromatic hydroxylation via P450 2C19. In the second binding pose, the methoxy group of the substrate is closer to the heme than the C_6_ atom: the C_Me_–O distance is 2.29 Å, while the C_6_–O distance is 5.17 Å. However, in binding poses 3, 4, 5 and 6, the C_6_ atom of the substrate is again closest to the heme, although in poses 4, 5, and 6 these two atoms are further than 4 Å from the heme. Consequently, binding poses 4, 5 and 6 may not be catalytically active poses, as the substrate is in the wrong orientation, with the normal metabolic sites pointing away from the heme. Moreover, the groups pointing toward the heme will be more difficult to activate. Nevertheless, the shortest substrate–heme interaction is found for binding pose 2 for the C_Me_–O interaction of only 2.29 Å, whereas the shortest C_6_–O distance is found in pose 1 at 2.95 Å. In all other poses, the substrate is well over 4 Å away from the heme. As the shortest distance between the substrate and heme was found for the C_Me_–O interaction, we decided to run molecular dynamics simulations from this structure and explore the substrate binding position and orientation as a function of time.

### 2.2. Molecular Dynamics Simulations

**Figure 5:**
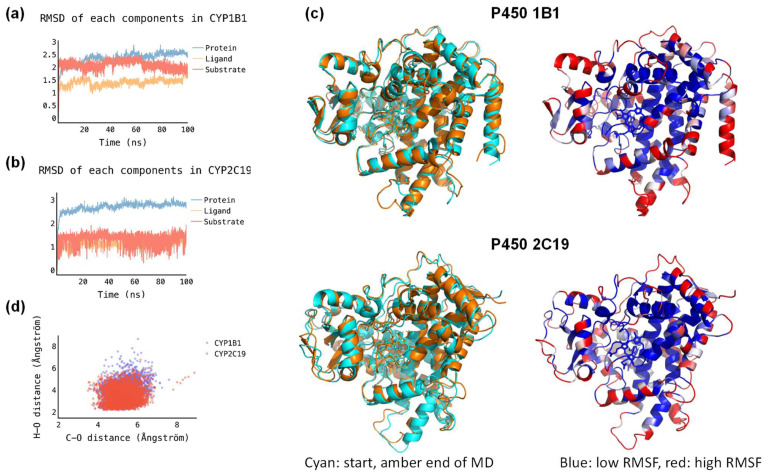
Data analysis of the MD simulations run on P450 1B1 and P450 2C19 with melatonin bound. (**a**) RMSD of P450 1B1 for the MD simulation over 5000 frames. (**b**) RMSD of P450 2C19 for the MD simulation over 5000 frames. (**c**) Snapshot analysis of P450 2C19 (in red) and P450 1B1 (in blue) MD simulations, where the substrate versus Cpd I distances are plotted for the C_6_–O and CH_2_H–O distances for each frame. (**d**) Overlay of the starting (in blue) and final (in amber) structures of the MD simulations for P450 1B1 (**top**) and P450 2C19 (**bottom**). Right-hand side gives the colored protein structures based on RMSF values during the MD simulations with blue representing small RMSF and red a large RMSF value.

Next, we selected docking pose 2 and ran a 200 ns molecular dynamics (MD) simulation in Amber for the P450 2C19 structure. In addition, a structure was created for melatonin-bound P450 1B1. As can be seen from Figure 5a,b the root–mean–square deviation (RMSD) of the P450 2C19 and P450 1B1 enzyme structures converge quickly (within several ns) and the RMSD stabilizes to a constant value. This applies to the RMSD of the protein chain, the heme ligand and the melatonin substrate during the MD simulation. As a consequence, it appears that both structures are highly rigid during MD simulations. To confirm this, we created an overlay of the starting and final structures of the two MD simulations, and show these in Figure 5c. Indeed, an overlay of the starting (in blue) and final (in amber) structures of the MD simulations for P450 1B1 and P450 2C19 provides an almost perfect match, with all chains and helices in approximately the same position and orientation. We also analyzed the root–mean–square fluctuations in the amino acid residues during the MD simulations, and show these in Figure 5c. Blue-colored residues show little movement during the MD simulation, while red-colored residues moved a lot. As can be seen, the inner residues around the heme and substrate binding pockets have low RMSF values, while the termini and helix end groups have large RMSF values. This is consistent with the tight and closed substrate binding pocket for both P450 isozymes.

We then analyzed the structures in more detail, and calculated distances between the oxo group of Cpd I with either the C_6_ atom of substrate (r_CO_) or to the nearest hydrogen atom of the methoxy group (r_OH_). For each snapshot from the two MD simulations, the distances r_CO_ and r_OH_ were measured and plotted against each other in a scatter plot; see Figure 5d. Thus, data points close to the *x*-axis have short O–H distances and are likely leading to methoxy group hydroxylation and subsequent deformylation, whereas data points close to the *y*-axis have short C_6_–O distances and have the substrate-binding orientation set-up for C_6_-hydroxylation instead. As can be seen from the scatter plot, most structures have the nearest methoxy hydrogen atom at a distance of Cpd I by 2–5 Å, whereas the C_6_–O distance is well over 4 Å in virtually all snapshots. The MD simulations, therefore, show that substrate is tightly bound in both P450 2C19 and 1B1 with relatively little flexibility and mobility. Moreover, the structural analysis indicates that the closest approach of substrate to heme is for the methoxy group, and hence the methoxy group will be the most likely point of activation for both isozymes. Indeed, experimental work on P450 2C19 showed that melatonin was the dominant methoxy group activation, with little or no aromatic hydroxylation products. The MD simulation for P450 2C19 confirms the product distributions that were experimentally obtained.

### 2.3. Quantum Mechanics Calculations

**Figure 6:**
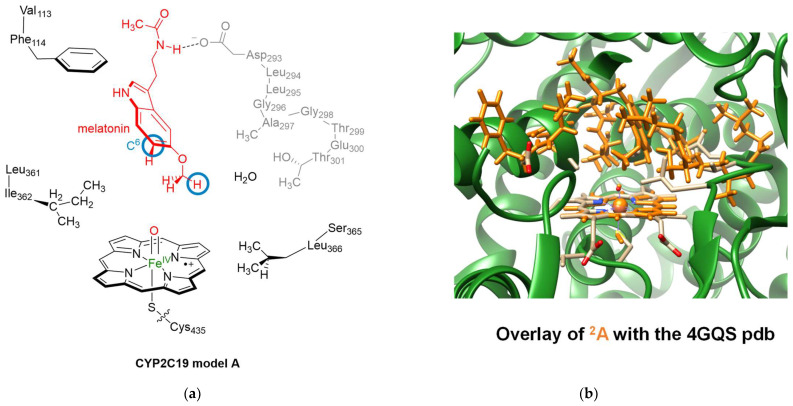
(**a**) Cluster model examined in the present study for the activation of melatonin by P450. Amino acids are annotated according to the 4GQS pdb file. (**b**) Superposition of the doublet spin-optimized geometry of **A** (depicted in amber) upon the crystal structure coordinates.

Subsequently, we constructed a model of P450 2C19 Cpd I with melatonin in the substrate binding pocket, as derived from the final snapshot of the MD simulation; see Figure 6: Model **A**. Cluster models were created using previously established procedures and include both the primary and secondary coordination spheres of Cpd I and the substrate [81,82,83]. As such, our model contains the heme, modified to protoporphyrin IX by substituting all side chains—including the propionates—with hydrogen atoms. This generally has only a minor effect on the electronic configuration and structure of the oxidant [84]. The axial cysteinate ligand (Cys_435_) was truncated to thiolate (SH^−^). Prior research indicated that thiolate offers more accurate electronic properties than methylmercaptane [85]. We positioned an oxo group adjacent to the iron at a distance of 1.63 Å, trans to the thiolate ligand. Melatonin, as the substrate, assumed the position of its most stable conformation based on the docking and MD simulations, with its methoxy group oriented towards the heme, while the amide group formed a hydrogen bond with the carboxylate group of Asp_293_. The model incorporated several peptide chains and groups, crucial in delineating the substrate binding pocket and facilitating hydrogen bonding interactions. Specifically, the chain spanning from Asp_293_ to Thr_301_ was incorporated, with residues Leu_294_, Leu_295_, and Glu_300_ simplified to a Gly residue. Additionally, peptide dimers including Val_113_-Phe_114_, Leu_361_-Ile_362_, and Ser_365_-Leu_366_ were embedded into the model. The complete model comprised 257 atoms and included one water molecule. Notably, the model was free from constraints.

**Figure 7:**
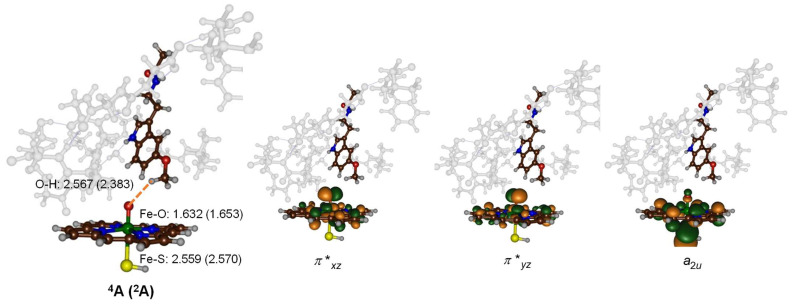
Optimized Cpd I geometries of P450 2C19 model A with DFT. Singly occupied molecular orbitals are also shown. Bond lengths are in Å.

Subsequently, geometry optimization of model **A** in both the doublet and quartet spin states was executed using density functional theory (DFT) approaches. The optimized geometry of ^2^**A** is presented in Figure 6 and superimposed on the crystal structure coordinates. As is evident, most protein chains retain similar positions in both structures, with the protein side chains oriented similarly. Details of the optimized geometries of ^2^**A** and ^4^**A** are depicted in Figure 7. The two spin state structures exhibit proximate energy levels. Considering the energy with zero-point energies (ΔE + ZPE), the doublet state is more stable than the quartet state by a margin of ΔE + ZPE = 0.2 kcal mol^−1^. However, this preference is reversed when thermal and entropic corrections and free energies are accounted for, leading to ΔG = −0.2 kcal mol^−1^. This narrow energy gap suggests that both states might coexist, implying potential reactivity across these two spin states [86,87,88]. The Fe–O bond length is relatively short, measuring 1.632 Å in the quartet spin state and 1.653 Å in the doublet spin state. Such bond lengths are consistent with previous calculations on P450 Cpd I models, whether derived from DFT methodologies on cluster models or quantum mechanics/molecular mechanics (QM/MM)-based calculations [89,90,91,92,93,94,95,96,97,98,99,100]. The iron-sulfur bond, involving two second-row elements, is long, as expected: 2.559 Å in the quartet state and 2.270 Å in the doublet state. In the optimized geometry, the methoxy group of the substrate is directed towards the heme and establishes a weak hydrogen bonding interaction between the C–H groups and the oxo entity of Cpd I. The O–H distances between Cpd I and the substrate are 2.567 Å for the quartet state and 2.383 Å for the doublet state. Consequently, the substrate is strategically positioned in the active site, primed for methoxy hydroxylation. This substrate alignment in the active site corroborates the experimental finding, which identified the exclusive *O*-demethylation of melatonin by P450 2C19.

The doublet and quartet spin states of Cpd I share the same orbital configuration, with each possessing three unpaired electrons situated in the π*_xz_, π*_yz_, and *a*_2u_ molecular orbitals. The molecular diagrams of these orbitals are provided on the right-hand-side of Figure 7. The π*_xz_ molecular orbital comprises a 3d_xz_ contribution from iron that establishes an interaction with the 2p_x_ orbital of the oxo group in an antibonding orientation. The corresponding bonding counterpart, the π_xz_ orbital, is energetically lower and doubly occupied. This results in a distinctive two-center-three-electron bond configuration within the *xz*-plane along the Fe–O axis. A parallel situation is seen in the *yz*-plane, where the π_yz_ orbitals are doubly occupied, while the π*_yz_ orbital remains singly occupied. These specific orbitals encapsulate the bonding and antibonding interactions between the iron 3d_yz_ and the oxygen 2p_y_ atomic orbitals. As illustrated in the orbital diagrams in Figure 7, the π*_xz_ and π*_yz_ molecular orbitals exhibit substantial mixing, with the π-orbitals stemming from the heme framework. Furthermore, Cpd I possesses a unique unpaired molecular orbital characteristic of the heme type, labeled as *a*_2u_ in D_4h_ symmetry. In the quartet spin state, this orbital contains an up-spin electron, whereas in the doublet spin state, it possesses a down-spin electron.

**Figure 8:**
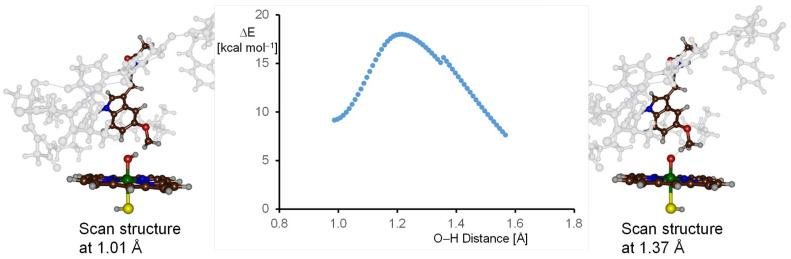
DFT calculated constraint geometry scan for hydrogen atom abstraction from the methoxy group by quartet spin Cpd I. Each data point represents a full geometry optimization with a fixed O–H distance. Several structures along the scan profile are shown.

Next, we initiated a hydrogen atom abstraction geometry scan of the optimized geometries of the quartet and doublet spin Cpd I structures (labeled ^4,2^**A**). An illustrative representation of the quartet spin landscape for this abstraction is presented in Figure 8. This scan was implemented by incrementally decreasing the O–H bond distance between the oxo group of Cpd I and the proximate hydrogen atom within melatonin’s methoxy group. Each step of the scan was marked by a comprehensive geometry optimization of the structure, maintaining a fixed O–H distance throughout. When we plotted these energies in relation to the initial energy of ^4^**A**, a characteristic bell-shaped curve emerged, with the reactants positioned on the right-hand-side of the scan and the products on the left. At the onset, there is an evident ascent in energy, which peaks and then gradually reduces, eventually giving rise to a complex for an iron-hydroxo and melatonin radical intermediates, collectively termed **IM1**. The uninterrupted curve signifies the structural integrity maintained during the scan, as no major geometrical changes are observed, especially in the second coordination sphere. We analyzed the structures on either extremity of the scan curve in detail and show these in Figure 8. In particular, the structure on the reactant side (O–H distance at 1.37 Å) and the structure on the product side (O–H distance at 1.01 Å) are highlighted. We observe analogous structural configurations for both the protein and heme components. The apex of the geometry scan is approximately 18.0 kcal mol^−1^ above the energy of the reactants. This position offers a suitable starting structure for a comprehensive transition state geometry optimization, which was subsequently performed. In addition, the lowest energy structure on the product-oriented side of the geometry scan curve serves as an initial structure for the full geometry optimization of the radical intermediate geometry. Moreover, the geometry scan provides evidence that the optimized structures of reactants, transition state and radical intermediates are directly connected with each other.

**Figure 9:**
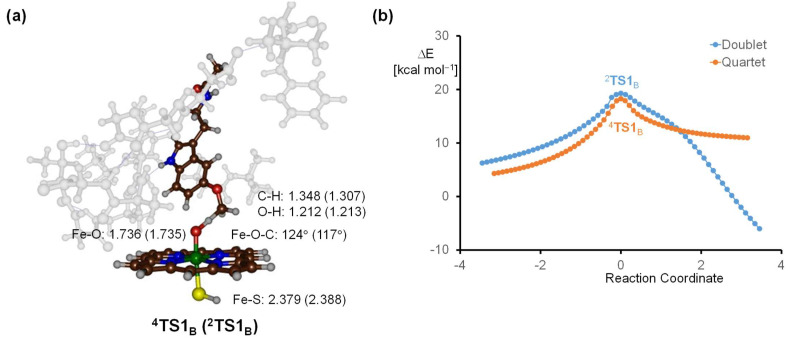
(**a**) DFT-calculated transition state geometry for hydrogen atom abstraction from the methoxy group by quartet and doublet spin Cpd I. Bond lengths are in Å and angles in degrees. (**b**) Intrinsic reaction coordinate profile for the reaction coordinate in forward and reverse directions, starting from the transition state geometry at a reaction coordinate of 0.

Subsequently, the structure, which corresponded to the highest-energy point from the geometry scan, was selected and subjected to a full transition state search. This led to the identification of a first-order saddle point, characterized by a single imaginary frequency that corresponds to the O–H–C stretch vibration (see Figure 9). In this transition state, the transferring hydrogen atom is situated midway between the donor carbon and the recipient oxo group. Specifically, for the structures ^4^**A** and ^2^**A**, the C–H bond distances are 1.348 Å and 1.307 Å respectively, while the O–H distances measure at 1.212 Å and 1.213 Å. The positioning of the transferring hydrogen atom indicates a structure leaning more towards the product side, as evident from the shorter O–H distance compared to the C–H distance. Upon the successful transfer of a hydrogen atom, there is a noticeable elongation of the Fe−O bond. In the quartet state, this bond lengthened from 1.632 Å (in ^4^**A**_B_) to 1.736 Å (in ^4^**TS1**_B_). Similarly, in the doublet state, the distance augmented from 1.653 Å to 1.735 Å (in ^2^**TS1**_B_). These geometrical transformations align well with prior computational results on hydrogen atom abstraction transition states facilitated by P450 Cpd I [99,100,101,102,103,104,105,106,107,108,109]. Such changes in bond lengths are anticipated, as the formation of the iron-hydroxo group results in the breaking of the π_xz_/π*_xz_ pair of molecular orbitals. In particular, the 3d_xz_ transforms into a nonbonding orbital that occupies one electron, while the other two electrons from the π_xz_/π*_xz_ orbitals form the σ_OH_ orbital of the hydroxo group [100]. Furthermore, the Fe–O–C angle between the iron-oxo group and the carbon of the methoxy group measures 124° for the quartet spin state and 117° for the doublet. These angles are in line with earlier calculations of hydrogen atom abstraction by P450 Cpd I, performed on minimal cluster models devoid of the second coordination sphere [101,102,103,104,105,106,107,108,109,110,111,112,113,114]. The structural features of the transition states match minimal cluster models, emphasizing that the substrate interacts with Cpd I in a nearly optimal orientation in P450 2C19. Moreover, this suggests that the protein environment has minimal influence on the substrate orientation and placement in P450 2C19.

When we look at the energetics of the reaction mechanism, it is clear that the transition states are higher than the reactants, as expected. Specifically, when we consider the energy values with zero-point correction, i.e., ΔE + ZPE, then the energy of ^2^**TS1**_B_ is 14.8 kcal mol^−1^ and ^4^**TS1**_B_ is 17.7 kcal mol^−1^ with respect to reactants. Not surprisingly, these values are close to the value of the maximum of the constrained geometry scan. As such, our geometry scan provided a good starting point for the transition state search and the scan provides an accurate representation of the energy profile. For another perspective on these barrier heights, it is worth mentioning the barrier of 17.8 kcal mol^−1^ energy calculated for the hydrogen atom abstraction from the terminal CH₃ group of *n*-propane, as described in a previous study [103]. As such, it appears that the abstraction of the hydrogen atom from the methoxy group of melatonin remains largely unperturbed by the effects of the second coordination sphere. An analysis of the second coordination sphere residues reveals that most residues from this sphere predominantly consist of aliphatic and aromatic amino acids. Their primary role appears to be anchoring the substrate and the oxidant in place. The sole noticeable hydrogen bond interaction between the protein and the substrate is from the amide group that interacts with the carboxylate of Asp_293_.

After the hydrogen atom abstraction transition states were optimized and characterized, we employed intrinsic reaction coordinate (IRC) scans for verification. This procedure followed the trajectory of the imaginary frequency to the nearest local minimum. The resulting IRC plots corresponding to ^2^**TS1**_B_ and ^4^**TS1**_B_ are shown in Figure 9b. These plots show that the reverse pathway of the IRC leads back to the initial state of Cpd I and the melatonin substrate, as expected. By contrast, the forward IRC pathway leads to the formation of the radical intermediate. The IRC scans, therefore, confirm the conclusions drawn from the geometry optimizations that the reaction between Cpd I and melatonin leads to a radical intermediate after hydrogen atom abstraction.

**Figure 10:**
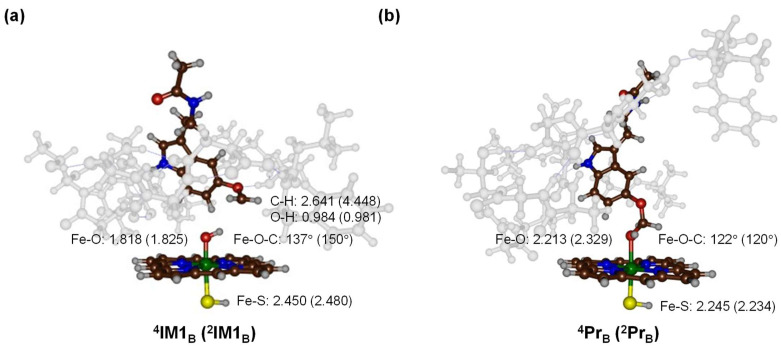
(**a**) DFT-optimized geometries of the radical intermediates **IM1**. Bond lengths are in Å and angles in degrees. (**b**) DFT-optimized geometries of the alcohol product complexes Pr. Bond lengths are in Å and angles in degrees.

After the hydrogen atom abstraction step and the formation of radical intermediates (**IM1**), we followed the reaction mechanism with a reaction step to form hydroxylated products (**Pr**). The optimized geometries for these species are shown in Figure 10. In the radical intermediate configurations, the Fe−O bond is elongated to 1.818 Å (^4^**IM1**_B_) and 1.825 Å (^2^**IM1**_B_). The Fe−S bond, on the other hand, remains considerably long. Electronically, both spin states show an unpaired spin density on the CH_2_ group of the substrate, with values of +1 and −1 for ^4^**IM1**_B_ and ^2^**IM1**_B_, respectively. This indicates that during the hydrogen transfer, an electron from the substrate transferred into the heme *a*_2u_ orbital. Both radical intermediates then possess a doubly occupied *a*_2u_ orbital, while the two π* orbitals remain singly occupied. The third unpaired electron is on the substrate and has up-spin in the quartet spin state and down-spin in the doublet spin state.

The rebound of the OH group from the heme to the radical was probed next. Interestingly, this process has no energy barrier and directly results in the formation of alcohol products (**Pr**). The process is highly exothermic, with energy values of ΔE + ZPE = −50.6 kcal mol^−1^ and ΔE + ZPE = −48.2 kcal mol^ࢤ1^ for ^4^**Pr**_B_ and ^2^**Pr**_B_, respectively. Such small rebound barriers suggest that radical intermediates ^4,2^**IM1**_B_ would be short-lived in nature, quickly transforming into product complexes. This fleeting existence ensures that there is minimal time for the radical or substrate to rearrange, which could lead to side reactions or alternate products. The resultant product complexes exhibit a weak interaction between the lone pair of the oxygen atom of the alcohol with the metal center, measured at a substantial distance for both ^4^**Pr**_B_ and ^2^**Pr**_B_. This interaction, being weak, would require minimal energy to disrupt, and allows for easy release of the product into the surrounding solution.

Finally, efforts were made to examine the C_6_ activation leading to aromatic hydroxylation. However, these attempts proved fruitless, as the geometry scan for bond shortening between C_6_ and oxygen resulted in collapse of the model and significant structural alterations that may not be realistic in an enzyme environment. In conclusion, these results imply that the substrate orientation and binding position, as well as the substrate binding pocket conformation, prevent C_6_-activation in P450 2C19 as they do not provide a favorable transition state that brings the substrate and oxidant close together.

## 3. Discussion

In this work, a computational study on P450 2C19 is reported, focused on understanding the product distributions that were experimentally obtained and how they compare to different P450 isozymes. We started with substrate docking, which led to a number of low-energy binding conformations. However, in most of these, the substrate is relatively far from the heme; hence, these docking sites may serve as secondary binding sites before the substrate latches in the final position prior to catalysis. The MD simulations show the substrate is tightly bound and stays in virtually the same position during a period of 200 ns. Moreover, the substrate is bound in an orientation that appears to prefer methoxy group hydroxylation rather than C_6_ activation, as the C_6_-O distance is relatively large in most snapshots, whereas the H−O distance to the methoxy group is much shorter. The MD simulations, therefore, have the substrate positioned and set up for methoxy group hydroxylation.

Subsequently, we created a large cluster model of 257 atoms and studied the methoxy hydroxylation mechanism with density functional theory approaches. The calculations show that the reactant Cpd I structure exists in close-lying doublet and quartet spin configurations, in agreement with previous DFT and QM/MM calculations of alternative P450 isozymes [9,29,33] that lead to two-state-reactivity patterns of competing doublet and quartet spin states. The rate-determining step is hydrogen atom abstraction, and a barrier of ΔE + ZPE = 14.8 kcal mol^−1^ for ^2^**TS1**_B_ and 17.7 kcal mol^−1^ for ^4^**TS1**_B_ was obtained. These barriers, particularly the doublet spin barrier, are lower in energy than those previously obtained for melatonin activation by P450 1A1 and P450 1A2. In particular, barriers of 17.8–22.3 kcal mol^−1^ (P450 1A1) and 16.5–18.8 kcal mol^−1^ (P450 1A2) were observed in these earlier studies [76,77]. As such, P450 2C19 has a better positioned substrate for methoxy group hydrogen atom abstraction than P450 1A1 and P450 1A2. Indeed, experimentally, P450 2C19 is known to lead to dominant methoxy group activation, in agreement with the lower hydrogen atom abstraction barrier. The barrier height for hydrogen atom abstraction is similar to values found for alternative substrates, such as the terminal methyl group of *n*-propane or propene [102,103] and, as such, the transition state for hydrogen atom abstraction from melatonin by P450 2C19 is not increased due to the stereochemical interactions with the protein environment.

The calculations presented here show that, after hydrogen atom abstraction, the structure relaxes to an iron–hydroxo complex that rapidly leads to OH rebound with a negligible barrier to form the alcohol products. It is expected that with the assistance of a proton from the solvent, deformylation occurs. We previously showed this step to be low in energy and not rate-determining [77]. Our calculations provide a good match with experimental product distributions from the literature and show that, after hydrogen atom abstraction, a fast rebound and facile deformylation leads to reaction products.

## 4. Materials and Methods

### 4.1. Enzyme Set-Up and Molecular Dynamics Simulations

An enzymatic P450 2C19 structure was created from the 4GQS pdb file, as taken from the Protein Databank [24,27]. The substrate and crystal water molecules were removed from the pdb file in Chimera UCSF [80] and chain A was selected. The heme was manually modified into a Cpd I structure, with an Fe−O bond length set to 1.63 Å. Hydrogen atoms were added to the structure using the Ambertools v1 package under pH 7 conditions [115]. We visually inspected key amino acid residues, and all Lys and Arg side chains were in the protonated states, while all Asp and Glu side chains were deprotonated. All histidine amino acids were in the singly protonated state. The melatonin structure was taken from our previous calculations [76] and converted into PDB format. Thereafter, melatonin was docked into the substrate-binding pocket using AutoDock Vina 1.2.0. [79] and binding pose 2 (see above) was selected for further studies.

MD parameters for the heme were created with the MCPB.py routine implemented in AmberTools 2018 [116], and then solvated in a rectangular box with a 10 Å distance between the box edges and the enzyme, and filled with TIP3P-defined water molecules. The ff14SB12 forcefield [117] was used for amino acid residues and protein structures. After the set-up, the system was minimized, and heated to 310 K with backboard constraints. Finally, a production run was performed for 200 ns without constraints.

### 4.2. Cluster Model Set-Up and Quantum Chemical Calculations

Based on the final snapshot of the MD simulation, we created quantum chemical (QM) cluster models of the first and second coordination sphere of the oxidant and substrate environment using previously reported approaches [81,82,83]. The P450 2C19 model is described as above, with protoporphyrin IX, all substituents truncated to hydrogen atoms and a central iron(IV)-oxo ligated to thiolate as the basic core. This system was supplemented with substrate, one water molecule, the amino acid side chains of the chain from Asp_293_ to Thr_301_, and the peptide dimers Val_113_-Phe_114_, Leu_361_-Ile_362_ and Ser_365_-Leu_366_. The amino acids Leu_294_, Leu_295_ and Glu_300_ were truncated to Gly residues, leading to a cluster model with an overall size of 257 atoms.

All calculations were run using the Gaussian-09 software [118] with density functional theory approaches. Previous validation and benchmarking studies of analogous systems showed that the methods and approaches used here reproduce experimental product distributions, reaction rates and selectivities very well [100,119,120]. The unrestricted hybrid density functional method B3LYP [121,122] was used for geometry optimizations, analytical frequency calculations and constraint geometry scans. The calculations utilized the effective core-potential-based LANL2DZ basis set on iron, and 6-31G* on the rest of the atoms [123,124], with the designated basis set as BS1. Single point calculations were carried out with cc-pVTZ on iron [125] and 6-311+G* on the rest of the atoms, with the designated basis set as BS2, to improve the energetics.

## 5. Conclusions

A comprehensive computational study on melatonin activation by P450 2C19 enzymes is reported. We initially conducted a molecular docking on a P450 2C19 structure and found several low-energy binding poses. We then selected the best conformation and ran a 200 ns MD simulation. The MD shows that the enzyme, and particularly the substrate binding pocket, are very tight and rigid, and show little movement during simulation. We then created a large cluster model of 257 atoms and studied substrate hydroxylation at the methoxy group and aromatic hydroxylation at the C_6_-position. The latter was not a feasible pathway for this enzyme due to the positioning of the substrate and oxidant, which prevented them from reaching close to C_6_. The aliphatic hydroxylation has a rate-determining hydrogen atom abstraction and barrierless rebound. The barriers match small model clusters of terminal alkyl group hydroxylation well. Consequently, substrate binding and orientation is set up for methoxy group hydroxylation and not aromatic hydroxylation. Our studies highlight the differences in substrate activation by various P450 isozymes and show that, in P450 2C19, the binding pocket is tight and guides the selectivity to deformylation as the dominant products.

## Data Availability

All data are fully available upon request from the authors.
